# *Providencia rettgeri* as an unusual pathogen in diabetic foot osteomyelitis: A case report and literature review

**DOI:** 10.1177/2050313X251395970

**Published:** 2025-11-20

**Authors:** Stephanie N. Campbell, Madeleine Mehaffey, Nikita Gambhir, Amanda L. Killeen

**Affiliations:** 1 School of Podiatric Medicine, University of Texas Rio Grande Valley, Harlingen, TX, USA; 2 Department of Orthopaedic Surgery, University of Texas Health Science Center at San Antonio, San Antonio TX, USA

**Keywords:** *Providencia rettgeri*, foot, wound, infection, osteomyelitis, diabetic foot osteomyelitis, multidrug resistance, snake bite

## Abstract

*Providencia rettgeri* is a Gram-negative bacterium, rarely reported as a cause of diabetic wound infections. Its frequent resistance to β-lactams and carbapenems limits treatment options, especially in immunocompromised patients. We present the case of a 59-year-old unhoused male living with diabetes, peripheral artery disease, and prior bilateral transmetatarsal amputations who presented with a University of Texas 3B diabetic foot ulcer. Magnetic resonance imaging showed osteomyelitis of metatarsals 2–4, and bone biopsy grew *Providencia rettgeri* and *Proteus mirabilis*. The patient declined further surgery and was treated with 6 weeks of cefepime and metronidazole, with outpatient wound care, achieving wound closure in 3 months. We performed a systematic scoping literature review on *Providencia rettgeri* wound infections in humans, detailed below, which identified only eight studies that met our inclusion and exclusion criteria. Most cases involved snake bites or critically ill patients, and none documented lower extremity bone involvement. Resistance patterns varied, but co-infection was common. Given its rarity and potential for multidrug resistance, *Providencia rettgeri* should remain on the differential for non-healing diabetic wounds. This case expands the limited literature and highlights the importance of culture-driven antibiotic therapy in complex infections. Recognition of *Providencia rettgeri* as a rare but clinically significant pathogen in diabetic foot osteomyelitis has implications for empiric antibiotic selection and clinical outcomes.

## Introduction

Diabetic foot ulcers (DFUs) are the leading cause of nontraumatic lower limb amputations.^1^ The global diabetes prevalence was 10.5% in 2021 and is expected to rise to 12.2% by 2045.^2^ This expected increase indicates more DFUs and their complications should be expected in coming years. Osteomyelitis is a frequent complication of DFUs, affecting >20% of moderate infections and 50%–60% of severe infections.^3^ It commonly leads to amputation and increases the likelihood of reulceration in the future. Specifically, reulceration after previous amputation due to DFU occurs in up to 40% of patients within 1 year.^1^ Treatment of osteomyelitis in the foot remains challenging, requiring long-term antibiotic coverage or, more commonly, amputation.^4^ Rising numbers of multidrug-resistant (MDR) organisms further complicate treatment by limiting antibiotic treatment options.

*Providencia rettgeri* is a Gram-negative, urease-producing bacillus, that is, ubiquitous in nature, commonly found in water, soil, and animal reservoirs. As an opportunistic pathogen, it is most often associated with polymicrobial urinary tract infections in patients with indwelling catheters.^5^ *Providencia* has also been reported in cases of pneumonia, meningitis, endocarditis, and septicemia.^6^ Although rare, wound infections account for <0.1% of reported cases, and osteomyelitis is even more uncommon.^7,8^ Of clinical concern is its frequent multidrug resistance, including the production of extended-spectrum β-lactamases and carbapenemases such as New Delhi metallo-β-lactamase-1.^9^ *Providencia* species exhibit intrinsic resistance to first-generation cephalosporins, aminoglycosides, tigecycline, and colistin (polymyxin E), agents often reserved for MDR Enterobacteriaceae.^6^ *Providencia stuartii* and *P. rettgeri* are typically implicated in nosocomial infections due to plasmid-mediated resistance mechanisms to β-lactamases, making susceptibility patterns a critical concern for microbiologists, infectious disease specialists, internists, and critical care teams. When these organisms are involved in wound or bone infections, their multi-drug resistance further complicates already challenging cases of chronic or non-healing wounds.

Recent reports, including cases from Morocco,^10^ Florida,^11^ and Saudi Arabia,^7^ have expanded the geographic scope of *P. rettgeri* wound infections and underscore its emerging clinical relevance beyond urinary isolates. Additionally, Hamed et al. described MDR *Providencia* isolates from DFUs in Egypt, which were later identified as a novel species (*Providencia pseudovermicola*), underscoring diagnostic challenges of *Providencia*.^12^

Several studies have shown clonal relatedness among isolates within the same hospital system, suggesting that *P. rettgeri* is often transmitted through nosocomial pathways.^13,14^ Given its rarity in wound and bone infection, coupled with multidrug resistance and propensity for hospital transmission, this case and literature review highlights the importance of identifying *P. rettgeri* early and tailoring antimicrobial therapy accordingly.

## Case report

Our case involved a 59-year-old African American male with a history of type 2 diabetes mellitus (HgA1c 7.3%), hypertension, hyperlipidemia, chronic kidney disease, peripheral artery disease, active smoking, and previous bilateral transmetatarsal amputations. The patient faced significant socioeconomic barriers to care, including being unhoused and living in a community shelter. He was admitted to a public safety-net hospital in a metropolitan area of southern Texas with a moderately infected (IWGDF/IDSA 3O), non-healing DFU to the distal end of his left transmetatarsal amputation stump (UT 3B).^15,16^ Laboratory studies showed sodium 140 mEq/L, potassium 3.2 mEq/L, chloride 110 mEq/L, calcium 8.4 mg/dL, glucose 101 mg/dL, albumin 2.7 g/dL, and white blood cell count 5.13 × 10^3^/µL. The patient did not meet SIRS criteria.^17^

**Figure 1.**
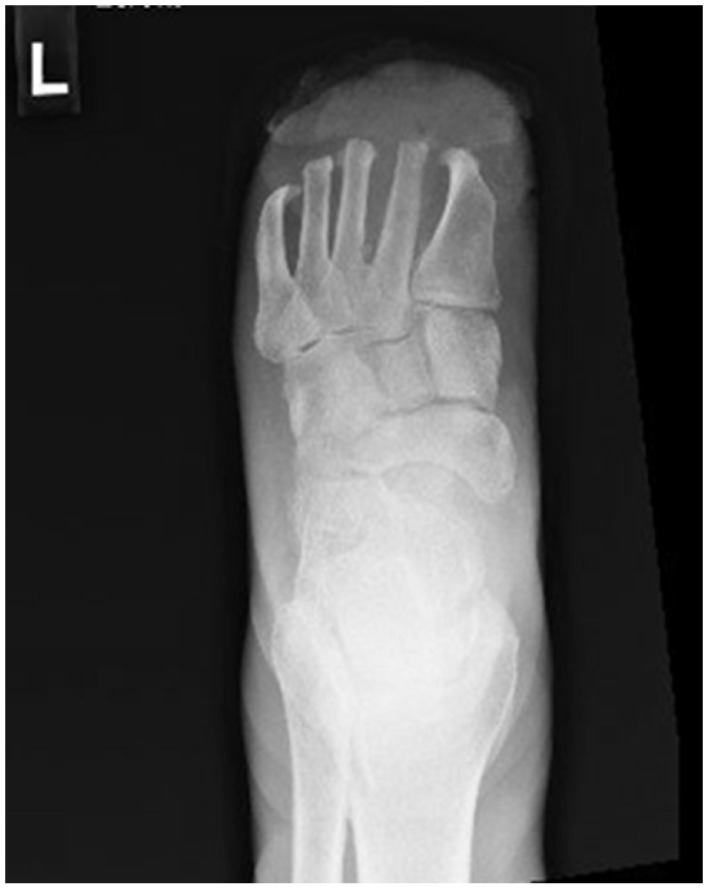
Left foot radiograph with soft tissue ulceration and periosteal reaction of the distal metatarsal shafts.

After radiographic assessment (Figure 1), magnetic resonance imaging was ordered to assess the presence and extent of osteomyelitis, which showed abnormal signal in metatarsal stumps 2–4 that extended proximally to the metatarsal bases.

Based on these results, combined with a history of amputation, the patient was offered resection of metatarsals 2–4, but he declined the procedure. An incision and drainage with intraoperative bone biopsy was performed. Blood and bone cultures grew *P. rettgeri* and *Proteus mirabilis*. The patient’s nares swab was positive for methicillin-resistant *Staphylococcus aureus*. The *P. rettgeri* strain cultured from our patient was resistant to ampicillin and cephalosporins but sensitive to cefepime and piperacillin/tazobactam (Table 1). Following the patient’s refusal of surgery, the focus shifted to optimizing medical therapy. The decision was made after multidisciplinary discussion with podiatry, infectious disease, and endocrinology teams. Social work was also consulted to address housing instability and limited access to care.

**Table 1.** Patient’s culture and sensitivity results.

Susceptible	Intermediate	Resistant
Amikacin	Cefpodoxime	Ampicillin
Ampicillin + sulbactam	Ceftriaxone	Cefazolin
Cefepime	Ertapenem	Ciprofloxacin
Cefotetan	Gentamicin	Trimethoprim + sulfamethoxazole
Meropenem	Tobramycin	
Piperacillin + tazobactam		

**Figure 2.**
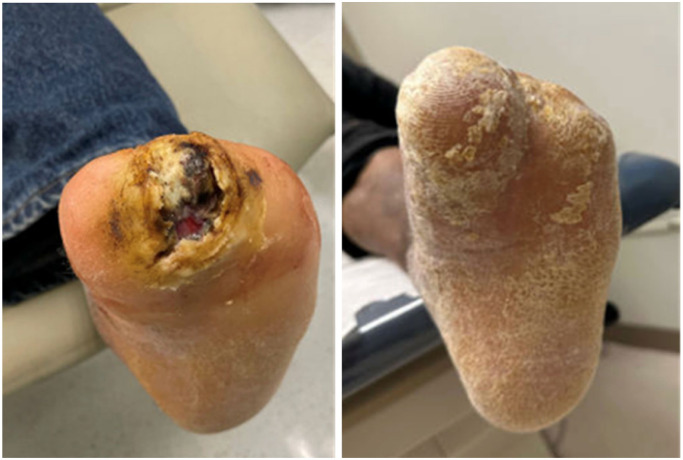
Clinical images during treatment and after wound closure.

Per infectious disease recommendations, the patient was started on 6 weeks of IV cefepime and oral metronidazole. He was followed bi-weekly in the wound clinic for 3 months after discharge. At each visit, the wound was evaluated for signs of superinfection or recurrence, and no reactivation was observed (Figure 2).

## Methods and literature review

**Figure 3.**
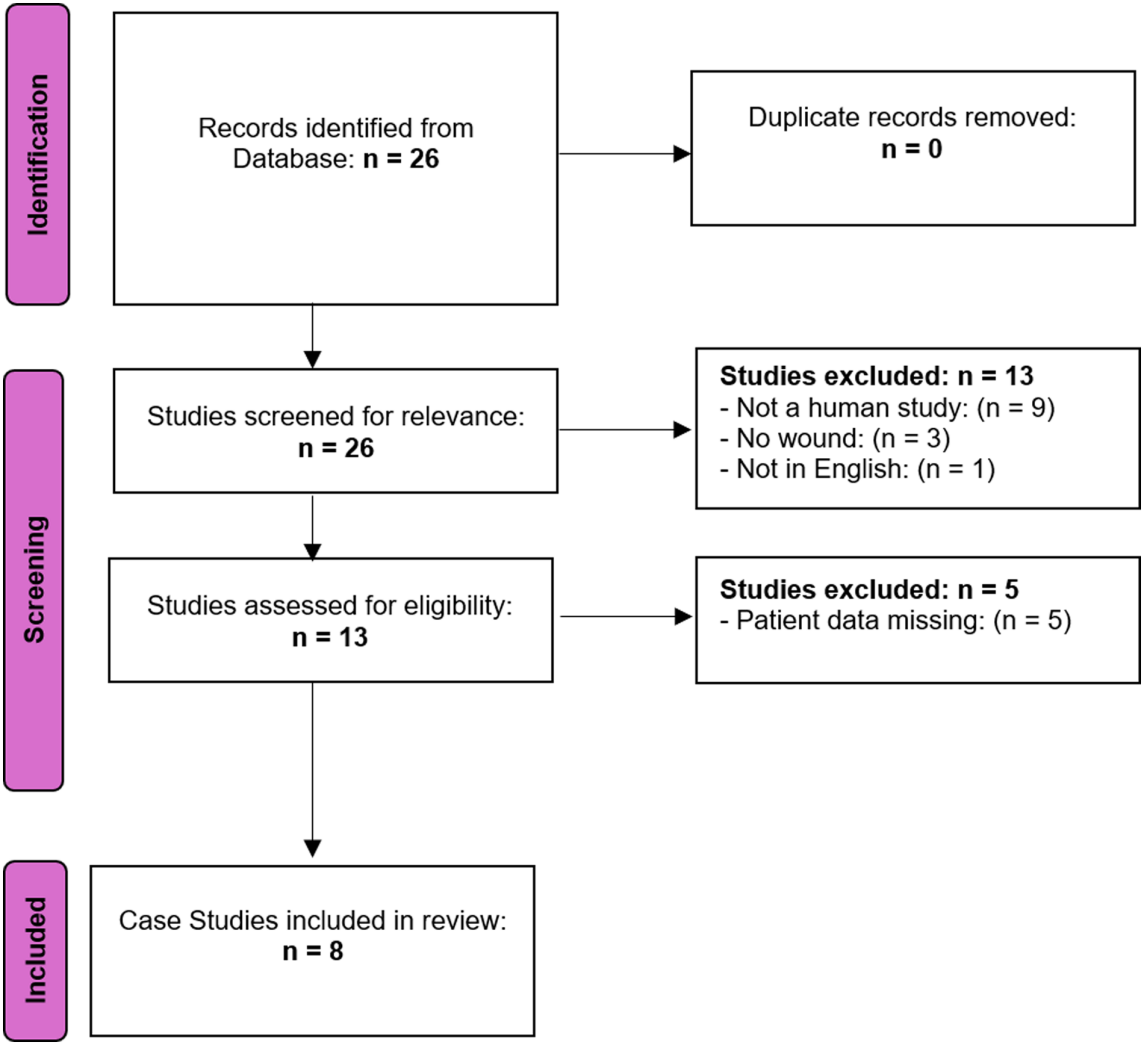
PRISMA flow diagram illustrating the selection process of sources of evidence for the scoping review.

To contextualize this case, we conducted a scoping literature review (Figure 3). PubMed was searched using the following keywords: “wound” AND “*Providencia rettgeri*” AND “diabetic foot,” which yielded no results. A broader search using “wound” AND “*Providencia*” AND “diabetic foot” identified one result describing *P. stuartii* and maggot therapy for osteomyelitis from 1997; the article was in Hebrew and not specific to our species. Refining the search to “wound” AND “*Providencia rettgeri*” yielded 26 results. We included only full-text articles in English, regardless of publication date, narrowing the selection to 13 papers. Inclusion criteria were human case reports or case series describing wound infections with *P. rettgeri*. Exclusion criteria included animal studies, urinary tract infections without wound involvement, or reports lacking clinical wound details. A total of eight papers met the criteria for final analysis.

**Figure 4.**
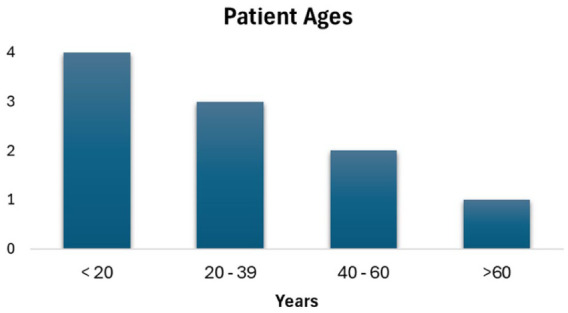
Age distribution of populations studied across the included sources in the scoping review. The chart illustrates the frequency and range of age groups affected by the pathogen of interest, *Providencia rettgeri.*

Table 2 summarizes the findings of our literature review. Cases were from various geographic locations, though most occurred in tropical or coastal climates. Notably, two studies described a cluster of cases due to snake bites, one with seven cases and the other with three. A third paper had two cases, and the remaining papers each reported single cases. Patient ages ranged from 4 to 72, with a predominance of infections in younger individuals (Figure 4). This age distribution contrasts with existing literature that identifies *P. rettgeri* as primarily affecting immunocompromised and elderly patients.^8,18^ Similarly, Abdallah and Balshi reported that carbapenem-resistant *P. stuartii and P. rettgeri* usually infect adult immunocompromised patients, with no pediatric patients in their review.^6^ This younger skew could be due to the over-representation of snake bites (10 out of 17 cases), which are more prevalent in children and adolescents in rural and tropical regions.^19^ The remaining cases occurred in high acuity settings such as burn units or intensive care units (ICU), reinforcing the existing literature’s description of *P. rettgeri*’s nosocomial, opportunistic nature.

**Table 2.** Characteristics and key findings of studies included in the scoping review.

Author	Number of patients	Patient age (years)	Geography	Infection location	Co-infections	Sensitivity	Resistance	Treatment/outcome
Jorge et al.^22^	7	NR	Brazil	Soft tissue abscess following snake bite	3 – *Morganella* 2 – *Streptococcus D*	AMK, Amino, CHL, CTX, Gent	TET, AMP	CHL healed
Washington et al.^28^	1	54	Guam	Anterior leg – ruptured tophi	*Staphylococcus aureus; Pseudomonas*	CIP, CRO, FEP, TZP	AMP, CFZ, Gent, SUL, TMP/SMX	CIP + Clinda, wound culture (−)
Tshisevhe et al.^21^	1	33	South Africa	Bedsore	HIV	None listed	Carb	Deceased
Otlu et al.^29^	1	35	Turkey	Burn	Not listed	COL, TGC	Carb, B lactamases	Not listed
Brenes-Chacón et al.^30^	3	7; 9; 12	Costa Rica	Soft tissue abscess following snake bite	1 – *Morganella; S. maltophilia*	None listed	None, AMP, Ceph, Gent	Not listed
Tchuinte et al.^31^	2	26; 63	Madagascar	Wound cultures, location unknown	Not listed	AMK, ETP, FEP, IMP, TZP	CAZ, CIP CTX, FOX, Gent, TMP/SMX, TOB, TZP	Not listed
Huff and Blome-Eberwein^11^	1	72	Florida, USA	Burn grafts on bilateral arms	MRSA, MSSA	AZT, FEP, CRO, Gent, MER, TMP/SMX, TZP	AMP, CFZ	*Empiric*: FEP, CFZ *Final*: LIN, CRO
Belmahi et al.^10^	1	4	Morocco	Calf	None	AMK, AZT, CRO	AMC, AMP, Carb, CFX, CHL, Gent, TMP/SMX	CRO healed

Amino: aminoglycosides; AMK: amikacin; AMC: amoxicillin–clavulanate; AMP: ampicillin; AZT: aztreonam; Carb: carbapenems; CAZ: ceftazidime; Ceph: cephalosporins; CFX: cefuroxime; CFZ: cefazolin; CHL: chloramphenicol; CIP: ciprofloxacin; Clinda: clindamycin; CRO: ceftriaxone; COL: colistin; CTX: cefotaxime; ETP: ertapenem; FEP: cefepime; FOX: cefoxitin; Gent: gentamicin; IMP: imipenem; LIN: linezolid; MER: meropenem; MRSA: methicillin resistant *Staphylococcus aureus*; NR: not reported; SUL: sulbactam; TGC: tigecycline; TMP/SMX: trimethoprim/sulfamethoxazole; TOB: tobramycin; TZP: piperacillin/tazobactam.

Antibiotic resistance was universally reported amongst all cases, though the profiles varied. The organism was frequently sensitive to ceftriaxone, cefepime, amikacin, and piperacillin/tazobactam. In contrast, resistance was seen to ampicillin, gentamycin, and carbapenems. As seen in Table 2, among those tested, four of four isolates were resistant to ampicillin, four of five to gentamycin, and three of five to carbapenems. Along with this resistance, 10 of 17 cases were reported as co-infections, with the remaining not specified.

All cases were soft tissue infections or abscesses; none reported bone involvement, particularly in the lower extremity. This makes our case of osteomyelitis arising from a DFU a unique contribution to the existing literature.

## Discussion

This review of *P. rettgeri* in human wound infections demonstrates that the organism is most often associated with immunocompromised patients, particularly those in burn units or intensive care settings, as well as in abscesses resulting from snake bites. Although less frequently isolated than other members of the Enterobacteriaceae family, *P. stuartii* and *P. rettgeri* are also notable causes of nosocomial infections, especially among hospitalized and ICU patients.^6,20^ *P. rettgeri* has been documented to be an important healthcare concern regarding hospital lines and catheter infections. In the paper by Tshisevhe et al., four cases were reported, three of which were excluded as they involved urinary, rather than wound, infections.^21^ All four patients were receiving dialysis, and three of them were complicated by HIV infections. The single case involving soft tissue infection resulted in death.

Soft tissue wound infections by this pathogen were rarely reported as monomicrobial. The most common co-infections reported in our literature review were *Morganella, S. aureus*, and *Streptococcus D.* The paper by Jorge reinforces this fact with all *P. rettgeri* cases being involved in co-infections.^22^ Our case aligns closely with the findings from the literature, as the patient had multiple risk factors for *Providencia* infection, including diabetes, history of ulceration and antibiotic use, homelessness, and immune compromise, with co-infection. Since the initial search was performed, Google’s search algorithms have changed, affecting which papers appear on the first page of search results. This may partially explain differences in article visibility or retrieval between searches performed at different times. To minimize this variability, we relied primarily on PubMed indexing and inclusion criteria to ensure consistency and reproducibility.

As demonstrated by our literature review, of particular concern is *Providencia*’*s* antibiotic resistance profile. Every case demonstrated resistance to at least one class of antibiotics, and the majority were classified as MDR. Clinicians should remain cautious when initiating empiric treatment for DFUs, particularly in complex or non-healing wounds, as organisms like *P. rettgeri* may be underrecognized and inherently resistant to common therapies. Wounds for which antibiotics reported to be effective in the reviewed cases included cefepime, ceftriaxone, amikacin, and piperacillin/tazobactam, aligning with previous recommendations for empiric therapy in polymicrobial or resistant Gram-negative infections.^23^ In a letter to the editor, a case of fourth toe osteomyelitis was described in a patient in Brazil with diabetes and traumatic burn. The isolate showed a similar resistance profile – resistant to carbapenem and meropenem, but sensitive to amikacin, gentamicin, and tobramycin.^24^ Early recognition through culture and sensitivity testing is critical beyond the expected typical and atypical agents preventing wound closure and successful treatment.

The primary limitation of this study is that it is a single case report, which inherently restricts generalizability. While clinical observations are valuable, they cannot establish standardized treatment. Our literature search is limited due to the rarity of *P. rettgeri* in diabetic foot osteomyelitis, introducing potential publication bias, where cases that are unusual appear more frequently in the literature while more common cases may be underreported. Variability in diagnostic methods and treatment protocols may further limit the ability to draw broad conclusions.

*P. rettgeri* osteomyelitis is so rare that searching PubMed for “*Providencia rettgeri*” AND “osteomyelitis” resulted in only two articles; one case in the frontoparietal bone of a patient post-neurosurgery and the other involving osteomyelitis in snakes. Grubb et al. reported a novel case of *P. rettgeri* osteomyelitis of the frontal bone following a snake bite and provided rationale that skull osteomyelitis is particularly life-threatening, with the patient ultimately going on to hospice care.^8^ Ramsay et al.’s group documented characteristic bony erosions in a ridge-nosed rattlesnake with *P. rettgeri* as the sole organism, cultured from the liver, heart, blood, and bone.^25^

In a retrospective single-institution review, Barshes et al. identified *P. rettgeri* and *P. stuartii*, combined, in only 0.9% of bone culture isolates in patients with DFUs, underscoring the rarity of these organisms in osteomyelitis.^26^ In summary, *P. rettgeri* demonstrates a low predilection for bone infection, making our case of diabetic foot osteomyelitis particularly noteworthy.

## Conclusion

*Providencia* species are opportunistic bacterial pathogens capable of causing a broad range of human infections, including soft tissue wounds and health care-associated line infections. While much of the literature to date has focused on isolates from developing countries, especially in the context of their notable antimicrobial resistance, the predilection for infection is often linked to medical devices.

This literature review suggests consistent resistance to carbapenems, including imipenem, ertapenem, and meropenem, underscoring a serious therapeutic challenge. Given the growing challenge of antibiotic resistance and the polymicrobial nature of diabetic foot infections, initial antibiotic selection should account for β-lactamase-producing organisms, particularly carbapenem-resistant Enterobacteriaceae. Clinicians must remain vigilant to the complexities of managing diabetic foot infections, which are further compounded by multidrug resistance and the emergence of under-recognized opportunistic pathogens. Close post-discharge monitoring is also essential in such cases to detect potential recurrence or superinfection. High-risk patients such as this are screened every 3–6 months for routine diabetic foot checks.^27^

Further research is needed to better characterize the pathogenic mechanisms of *Providencia* in bone and deep tissue infections, including its interaction with co-infecting organisms and host immune responses. Larger case series and molecular studies could help clarify resistance mechanisms, guide empiric therapy, and inform strategies for prevention, particularly in high-risk populations.
